# The Effect of High-Intensity Ultrasound and Natural Oils on the Extraction and Antioxidant Activity of Lycopene from Tomato (*Solanum lycopersicum*) Waste

**DOI:** 10.3390/antiox11071404

**Published:** 2022-07-20

**Authors:** Gieraldin Campos-Lozada, Xóchitl Alejandra Pérez-Marroquín, Graciela Callejas-Quijada, Rafael G. Campos-Montiel, Alejandro Morales-Peñaloza, Arely León-López, Gabriel Aguirre-Álvarez

**Affiliations:** 1 Instituto de Ciencias Agropecuarias, Universidad Autónoma del Estado de Hidalgo, Av. Universidad Km 1, Tulancingo C.P. 43600, Hidalgo, Mexico; ca409778@uaeh.edu.mx (G.C.-L.); pe409780@uaeh.edu.mx (X.A.P.-M.); ca290659@uaeh.edu.mx (G.C.-Q.); rcampos@uaeh.edu.mx (R.G.C.-M.); arely_leon@uaeh.edu.mx (A.L.-L.); 2 Escuela Superior de Apan, Universidad Autónoma del Estado de Hidalgo, Carretera Apan-Calpulalpan s/n, Colonia Chimalpa Tlalayote, Apan C.P. 43920, Hidalgo, Mexico; amorales@uaeh.edu.mx; 3 Uni-Collagen S.A. de C.V. Arnulfo González No. 203, El Paraíso, Tulancingo C.P. 43684, Hidalgo, Mexico

**Keywords:** tomato waste, lycopene, antioxidants, high-intensity ultrasound, green technology

## Abstract

The extraction of lycopene was carried out with three types of vegetable oils (grape, extra virgin olive, and peanut) by means of two methods: agitation and high-intensity ultrasound with a frequency of 20 kHz at an amplitude of 80% with periods of 40 s of sonication for 20 min at a temperature of 40 °C. The antioxidant determination by inhibition of ABTS and DPPH radicals showed no significant differences (*p* > 0.05) for inhibition of the ABTS radical in native oils and oils with lycopene. However, the radical DPPH showed that the native oils presented significant differences (*p* ≤ 0.05) compared to the samples with lycopene. FTIR spectra revealed the characteristic functional groups of lycopene exhibiting two characteristic peaks at 2923 cm^−1^ and 2957 cm^−1^. The DSC thermograms showed that the higher the degree of oil unsaturation, the lower the melting temperatures. Olive oil was the least unsaturated with the highest amount of oleic fatty acid. Grapeseed oil reported the lowest melting temperature at around −24.64 °C. Extra virgin olive oil showed the lightest values (L* = 41.08 ± 0.45) of brightness, and the peanut oil with lycopene was the darkest (L* = 16.72 ± 0.05). The extraction of lycopene from organic wastes treated with agitation and ultrasound was satisfactory reducing the use of conventional solvents. However, extraction with olive oil under agitation showed the best results.

## 1. Introduction

Tomato is a herbaceous plant species belonging to the *Solanum* genus of the *Solanaceae* family [1]. This fruit is essential in the basic food basket. It is grown in greenhouses or in the open field. The quality of tomato decreases significantly due to mechanical damage, incorrect transportation, and storage time [2]. It is estimated that 10–15% of the total volume is considered waste and it is commonly used for compost or animal feed. Tomato is a very important fruit in the human diet and its consumption could be natural or cooked. It is rich in antioxidants and is considered an important source of carotenoids and lycopene [3].

The *Solanum lycopersicum* tomato is one of the most consumed tomatoes in the world, mainly for its nutritional value and its use as an ingredient in various foods. For the processing of tomato derivatives, the skin and seeds (pomace) are usually discarded. This by-product represents between 1.5% and 5% of the initial weight. Currently, this percentage of the waste is considered important for the industry as it contains important compounds. The peel has a higher amount of fiber, carotenoids, and phenols, while the skin is mainly composed of oil and proteins [4].

Lycopene, one of the main carotenoids present in tomatoes has several beneficial properties and acts as a potent antioxidant, protecting cells against oxidative damage and thereby reducing the risk of chronic diseases [5]. Other studies mention that this compound is an ingredient with many positive contributions to human health and physiology [6]. It is a hydrophobic antioxidant that confers the red color pigment characteristic of tomatoes and increases with ripening [7]. It also protects human cells from oxidative stress caused by the action of free radicals which are responsible for aging, cardiovascular diseases, and various types of cancer [8]. This antioxidant is considered a potent antioxidant with high anticarcinogenic and radical scavenging activity, and acts as a potential protector against prostate cancer, although this information still has limited data [9]. This carotenoid is characterized by a chemical structure with an open aliphatic chain formed by 40 carbon atoms with 13 double bonds (11 are conjugated). Its structure is highly reactive with oxygen and free radicals [10]. Its molecular weight is 536.85 g mol^−1^. It is insoluble in water and soluble in some solvents [11]. Tomato possesses 90% *trans* isomers, once it is processed, it contains more *cis* isomers due to thermal treatments. The *cis*-isomeric forms identified in lycopene are: 5 *cis*, 9 *cis*, 13 *cis*, and 15 *cis* [12]. The type of solvent to extract plant compounds is important since low toxicity solvents must be used. The solvent must have the capacity to absorb and transmit the energy of the ultrasonic wave and penetrate the interior of the material to extract the bioactive compounds. The polarity of the solvent also influences the improvement of the extraction [13]. Previous studies showed that edible vegetable oil has high potential as a solvent in the ultrasound extraction of desired compounds due to its convenience and ability to be enriched with carotenoids [14].

Several research groups are focused on the extraction of lycopene by using various techniques, e.g., conventional solvents [15], through cellulolytic and pectinolytic enzymes [16], supercritical carbon dioxide (SC-CO_2_) [17,18,19]. However, alternative techniques are currently investigated to obtain an optimum environmentally friendly and of reliable method extraction to extract this carotenoid [20]. The main objective of this paper was the extraction of lycopene from tomato (*Solanum licopersicum*) and its characterization of its physicochemical, thermal, antioxidant and viscosity properties by using emerging technology such as high-intensity ultrasound.

## 2. Materials and Methods

### 2.1. Materials

Tomato (*Solanum lycopersicum*) wastes at their highest state of ripeness were collected from a market center of Tulancingo de Bravo, state of Hidalgo, Mexico. Three types of oils were used as extractive substances: extra virgin olive (San Miguel), grape (enature), and peanut (enature). The reagents used for antioxidant activity such as 1-Butanol and methanol were of analytical grade obtained from JT. Baker (Fisher Scientific SAS, Lenexa, KS, USA). The treatments were described with the following keywords: Natural oils: extra virgin olive oil (O), grape oil (G), and peanut oil (P). While the oils with lycopene were identified as follows: OL, GL, and PL, respectively.

### 2.2. Lyophilization of Tomato

The Lyophilization process was carried out according to the methodology described by Gümüsay et al. with some modifications [21]. Sliced ripe tomatoes were placed in an ultra-freezer at −80 °C. Once this temperature was reached, the tomato was placed in a Labconco freeze-dryer at −40 °C and 2 mBar (Freeze Dryer Model 79480 LABCONCO, Kansas City, MO, USA). Then, tomatoes were ground in a blade mill (Retsch GM 200, Haan Alemania) for 10 s at 10,000 rpm. Tomato powder was finally stored in a dark environment at −4 °C. 

### 2.3. Extraction Process

#### 2.3.1. With Ultrasound Application

Extraction of lycopene was carried out according to Rahimi with some modifications [22]. A total of 50 mL of oil were measured and placed in a 100 mL beaker covered with aluminum foil, 10 g of lyophilized tomato were weighed and added little by little to the beaker containing the oil. The sample was homogenized to reach an oil/tomato concentration of 5:1 (*w*/*v*). Sample was sonicated in a high-intensity ultrasound (SONICS Vibra-cell 130 watt) with the following settings: 20 kHz frequency, 80% amplitude for 20 min pulsations of 40 s and intervals of 20 s. The sonication increased the temperature to around 40 °C. To avoid sample overheating, the container was placed into another container with ice to keep the temperature around 10 °C. At the end of the sonication time, the sample was centrifuged in a (Z 36 HK centrifuge, Hermle, Germany) for 10 min at 4 °C to achieve the separation of oils. Finally, the supernatant (lycopene with oil) was placed in amber vials and kept at room temperature. 

#### 2.3.2. Extraction with Agitation

10 g of freeze-dried tomato and 50 mL of each of the oils were weighed. The tomato and oil were placed in a 100 mL beaker covered with aluminum foil; inside the beaker, a magnetic stirrer was placed. The beaker was placed on a Corning brand stirring rack followed by 17 h of agitation at room temperature.

### 2.4. Determination of Antioxidant Activity

For antioxidant activity measurements, 1 mL of the sample consisted of oils and lycopene were diluted in 5 mL of methanol:1-butanol (1:4) (Sigma-Aldrich; St. Louis, USA) to dissolve correctly the oily sample into the solvent. The ABTS (20-azino-bis [3-ethylbenzothiazoline]-6-sulfonic acid) (Sigma-Aldrich; St. Louis, MO, USA), measurements were carried out based on the Re et al. methodology with some modifications [23]. ABTS was stabilized with the solvent mixture until the reading on the spectrophotometer (Jenway Genova, Model 6705, Bibby Scientific; Staffordshire, UK) was 0.70 ± 0.02 at 734 nm. A total of 200 μL of this diluted sample were placed in test tubes previously covered with aluminum to protect them from light sources. Then, 2 mL of stabilized ABTS radical were added and stored for 7 min. The DPPH radical (2,2-Diphenyl-1-picrylhydrazyl) was obtained from Sigma-Aldrich; (St. Louis, MO, USA). The methodology described for Brand-Williams and collaborators [24] was followed with some modifications. A total of 500 μL of diluted sample and 2.5 mL of stabilized DPPH radical were prepared and stored for 30 min to allow the reactions between DPPH and sample. The readings were taken at 515 nm. 

### 2.5. Color Determination

Lycopene color was determined as described by Stinco with some modifications [25]. A total of 5 mL of each sample were placed in a white container and analyzed using the CieLab scale by means of a colorimeter, model CR-400/410 (Konica Minolta, Foster City, CA, USA), three replicates were considered for each sample.

### 2.6. Microscopic Visualization of Tomato Lycopene 

Samples were prepared as described by Rahimi [22] with some modifications. The tomato in its fresh and natural state was cut in half in order to obtain a small fragment of pulp. Samples were placed on an object holder to visualize the cells. Sample was covered with a coverslip and placed in a microscope (Olympus CX31RBSF, AMERICA North (USA-Canada-Mexico)) with a 100× objective. 

### 2.7. Fourier Transform Infrared Spectroscopy (FT-IR)

Absorption spectra from the freeze-dried samples were obtained by Fourier transform infrared spectroscopy (FTIR) (Perkin Elmer; Boston, MA, USA) equipped with attenuated total reflectance (ATR). This technique was based on the methodology described by Rahimi and M. Mikani with some modifications [22]. Samples were placed in intimate contact with the diamond crystal by applying a loading pressure and scanned in a range of 400 cm^−1^ to 4000 cm^−1^ at room temperature. The results were analyzed with Spectrum TM 10 software (Perkin Elmer, Boston, MA, USA). 

### 2.8. Differential Scanning Calorimetry (DSC)

Thermal stability was analyzed by using differential scanning calorimetry (DSC) series Q 2000 with an intracooler RCS90 (TA Instruments; New Castle, DE, USA). The melting temperature (Tm) and enthalpy (ΔH) were calculated. An empty standard aluminum tray (TA instruments) was used as a baseline. A total of 1.5 ± 0.1 mg of the sample were placed in hermetically sealed aluminum trays and scanned in a range of 25 °C to 150 °C with a heating rate of 10 °C per minute. The values of Tm and ΔH were evaluated with the software TA 2000 analysis software (TA Instruments; New Castle, DE, USA). These parameters were calculated based on endothermic changes recorded in the thermogram.

### 2.9. Ultraviolet-Visible Spectroscopy (UV-Vis)

The spectra of the samples were obtained with the Lambda 45-UV-VIS ultraviolet-visible spectrometer (Perkin Elmer. Waltham, MA, USA) equipped with a quartz cell (optical path length: 1 cm) at room temperature [26].

### 2.10. Statistical Analysis

The experiment was conducted as a completely randomized design with three replicates. Data were analyzed by one-way analysis of variance (ANOVA), and means were separated using the Tukey test with the SPSS software (SPSS Inc., Chicago, IL, USA) v25. A significance value of α = 0.05 was used to distinguish significant differences.

## 3. Results

### 3.1. Effects of the Inhibitor Used in Antioxidant Activity Test

Antioxidant agents have been classified by their mechanism of action as either primary or secondary. Primary antioxidants, also known as “chain-braking”, are mainly phenols, carotenoids, flavonoids, tocopherols, and tocotrienols which are present in vegetables. These inhibit or retard lipid oxidation by donating hydrogen atoms to the radicals. Chain-braking have a higher affinity for peroxyl radicals than for lipids. Secondary antioxidants, known as “preventers”, act by inhibiting initiation through chelation of metal ions mainly copper and iron. These are also known to reduce the rate of lipid oxidation through singlet oxygen deactivation, transition mechanisms, regeneration, UV absorption, and oxygen scavenging [5]. 

Tomato lycopene is considered a secondary antioxidant that prevents the oxidation of free radicals and lipid oxidation. The determination of antioxidant activity was carried out by using ABTS (20-azino-bis [3-ethylbenzothiazoline]-6-sulfonic acid) and DPPH (2,2-diphenyl-1-picrylhydrazyl) inhibitors. ABTS is used to detect antioxidant activity in hydrophilic and lipophilic media [27], while the DPPH radical is among the most stable and used with the best results in lipophilic media [28]. 

**Figure 1:**
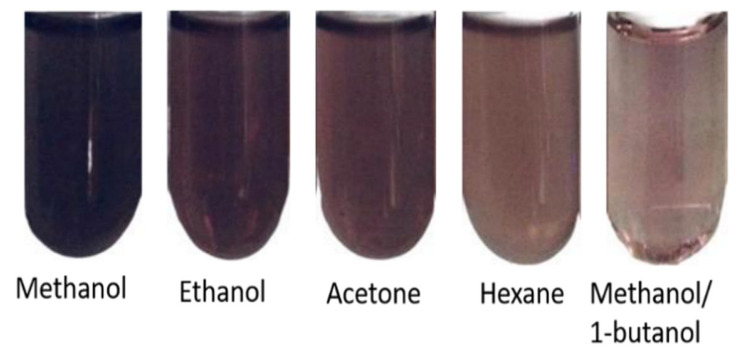
Degree of turbidity on the reaction of mixture solvent-lycopene and DPPH radicals.

Before the antioxidant activity determination, different mixtures of solvents were evaluated in order to identify the most compatible with the sample based on the formation of turbidity. Lycopene was dissolved in methanol/1-butanol in a ratio of 1:4, hexane, acetone, and ethanol. Samples prepared with oils and oils with lycopene dissolved very well with solvents hexane, acetone, and ethanol. However, these samples presented turbidity when in contact with the radicals, generating a problem for a good determination of the antioxidant activity. The best solvent resulted in the mixture of methanol/1-butanol. This sample remained translucent allowing the determination of antioxidant inhibition and a better reading in the spectrophotometer as shown in Figure 1.

Once the methanol-1-butanol was identified as the best solvent with less turbidity, the determination of antioxidant inhibition was performed. 

**Figure 2:**
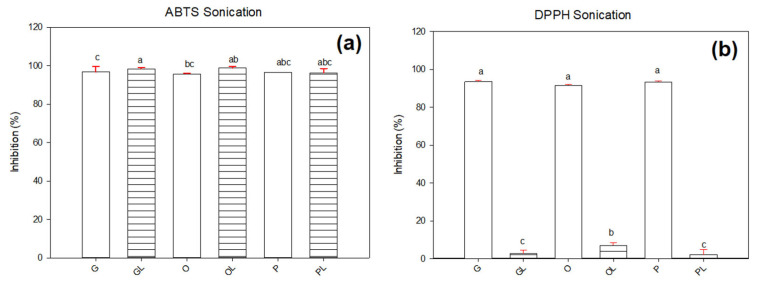
Radical inhibition in native oils and oils with lycopene by sonication technique using ABTS radical (**a**,**b**) DPPH radical. Letters (a–c) represent significant differences between samples.

Figure 2 shows the percentage of ABTS and DPPH radical inhibition in samples subjected to sonication. The ABTS (see Figure 2a) showed significant differences (*p* ≤ 0.05) between native oils and oils with lycopene. The higher inhibition range appeared in the sample GL with 98.3%. However, this particular sample resulted in lower inhibition when measured in G with 96.6%. For samples O and OL, the ABTS inhibition resulted in 95.7% and 98.8% respectively. It can be noted that the presence of lycopene in this sample increased the antioxidant activity. However, the samples P and PL showed no differences (*p* ≥ 0.05) with inhibitions around 96.5% and 96.1% respectively. 

The results for the inhibition of the DPPH radical shown in Figure 2b show that the DPPH inhibitor resulted more efficient to detect the antioxidant activity. Native oils G, O, and P resulted in higher (93.5%, 91.5%, and 93.4% respectively) values of inhibition compared with those including lycopene GL, OL, and PL (26%, 6.9%, and 2.1% respectively). Previous results reported inhibition of the DPPH radical of 50% in a study of lycopene extracted from tomato waste assisted with ultrasound and sunflower oil as solvent [22]. In another study of antioxidant activity performed in tomato jelly beverages, about 50% of DPPH free radical inhibition was achieved. This study was performed by extracting carotenes with organic solvents [29]. Oxidation in lipids during the rancidity period produces free radicals that generate undesirable tastes and odors, one of the techniques to measure the oxidation of a lipid is by inhibition of the DPPH radical (2,2-Diphenyl-1-picrylhydrazyl). 

Based on the above results obtained from samples subjected to sonication, a comparison was made with results from samples subjected to agitation without sonication. This comparison was carried out to evaluate the antioxidant activity by means of another technique and thus to have a broader view to study the antioxidant behavior of oils and extracts with oil.

**Figure 3:**
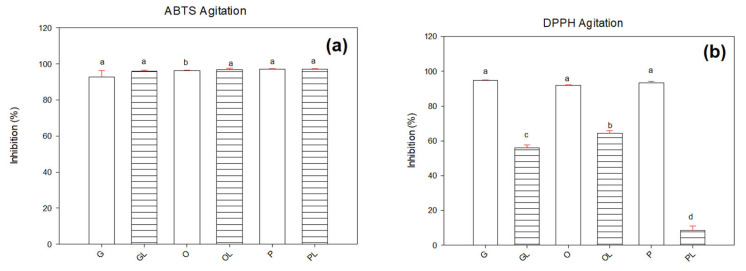
Inhibition of radicals in native oils and oils with lycopene by the agitation technique using (**a**) ABTS radical and (**b**) DPPH radical. Letters (a–d) represent significant differences between samples.

Figure 3 shows the percentage of ABTS and DPPH radical inhibition in samples subjected to agitation. The ABTS results (see Figure 3a) showed no statistical differences (*p* ≥ 0.05) in all the samples evaluated with values in the range of 95 to 96%. 

However, when the DPPH radical was determined (see Figure 3b), it was observed that the tree native oils G, O, and P inhibited the radical between 91 and 94% and there were no significant differences (*p* ≥ 0.05) between them. 

Samples with lycopene resulted in a decrement in inhibition properties with respect to native oils. OL sample resulted with 64%, followed by GL with 55%, and the sample PL showed the lowest inhibition with 8%. These results can be attributed to the fact that DPPH radical shows more affinity to analyzing lipophilic samples. DPPH was the most effective radical to assess antioxidant activity in both agitation and sonication samples. This could be due to the fact that sonication may generate the oxidation of lycopene. 

### 3.2. Comparison of Sonication vs. Agitation Technique on the Antioxidant Activity

**Figure 4:**
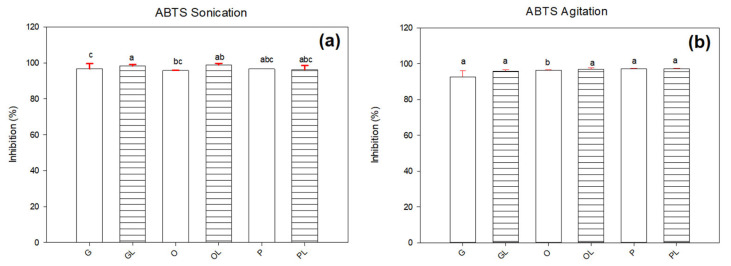
ABTS radical inhibition in samples subjected to (**a**) sonication and (**b**) agitation. Letters (a–c) represent significant differences between samples.

**Figure 5:**
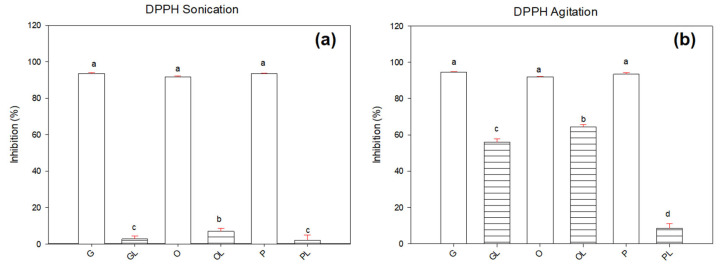
DPPH radical inhibition in samples subjected to (**a**) sonication and (**b**) agitation. Letters (a–d) represent significant differences between samples.

The extraction of lycopene in an oily medium is influenced by several factors. In order to analyze the data from another perspective, the same results were analyzed from a point of view of the extraction method. According to Figure 4, the ABTS radical showed incompatibility with samples no matter what the extraction method was. The average values of inhibition in both techniques ranged between in the range of 95–98%. However, for the DPPH radical (see Figure 5), it was observed that there were significant differences (*p* ≤ 0.05) in native oils and oils with lycopene. Based on these results, there were detected higher values of inhibition in samples treated under the agitation method. It means that the sonication process reduced significantly the antioxidant activity of samples containing lycopene. The GL sample showed 55.9% and 2.6% for agitation and sonication techniques, respectively. The most significant reduction of antioxidant activity due to the sonication process was observed in the OL sample with 64.3 to 6.9%. This significant reduction in inhibition could be due to the formation of free radicals due to cavitation. Ultrasound extraction of lycopene could be affected by the cavitation phenomenon due to the rupture of tomato membranes. It releases the lycopene from the chromoplasts of the cell. In some compounds, cavitation generates free radical formation due to the elevated temperatures and the high energy released by the probe. Azuola and Vargas [30] mentioned that reducing the particle size increases the area of exposure to the solvent and the cavitation. The strong collapses that occur during transient and repetitive cavitation can generate high temperatures at a localized level (greater than 5000 K). This causes chemical changes in the vapor phase within the cavitation bubble and in the immediate surrounding fluid. Cavitation generates microscopic bubbles that collapse and can form free radicals. The increase in these molecules containing an unpaired electron could have generated a decrease in lycopene concentration [31]. This could be the reason why samples GL, OL, and PL may be oxidizing due to the formation of free radicals generated by cavitation and the release of metals. The presence of conjugated double bonds of lycopene could turn the sample unstable [32]. Rawson et al. [33] reported that the determination of time, temperature, pulsations, and solvent/material ratio are important factors to obtain a good extraction of lycopene. There may also be a degradation of lycopene because the mentioned parameters modify chemical, physical, and biological processes that can affect the final product. Hernandez and collaborators [34] studied the effect of temperature on the accumulation of tomato carotenoids at different stages. They concluded that stress caused by high temperatures causes an alteration in the composition of the fruit. The processing of tomatoes to 32 °C causes a degradation of lycopene and other carotenoids and β-carotene are formed due to the mechanisms of control and metabolic channeling of isoprenoids that determine the final concentration of each compound. Cardona and Restrepo [35] reported that lycopene should be subjected to a temperature lower than 60 °C to avoid degradation. Other studies mention that tomato processing can increase the amount of lycopene, the optimum temperature during lycopene heating is 70 °C taking care that the temperature is not above this value to avoid loss of lycopene. They also mention that temperature and mechanical operation weakens the binding force between lycopene and the tissue matrix facilitating the rupture of cell walls and achieving the release of lycopene [29]. For the reaction of two or more compounds, it is preferable to be in contact and in agitation. This technique is widely used because it is one of the most economical. Stirring generates a homogeneous medium in the liquid phase by means of a dilution that facilitates its contact. One of the most commonly used agitation techniques is magnetic, based on the rotation of an external magnetic field that rotates a magnet introduced into a solution [36]. It is considered that these results could be due to the fact that the agitation technique is less aggressive than the sonication technique. In addition, there is no presence of additional radicals generated by the ultrasonic probe.

### 3.3. Interaction of Native Oils with Lycopene

Lycopene is one of the most studied carotenoids due to its high content present in tomatoes. It is very stable in its natural environment. However, when it is heated, purified, or extracted with oils or organic solvents, it becomes unstable because some of its constituent substances can make structural modifications and even destroy pigments. These pigments can decrease their coloration due to the loss of conjugation of the molecule in the region of the double bonds, but not to the degree of breakage of the hydrocarbon skeleton [37]. Lycopene as a carotenoid is known to have a higher capacity to inactivate photoactive sensitizers that form singlet oxygen. It is a material containing 13 double bonds, 11 of which are conjugated. These double bonds can be easily attacked by electrophilic reagents resulting in high reactivity towards oxygen and free radicals. The highly lipophilic nature of lycopene has a higher antioxidant activity at the cell membrane level by interacting with lipid components [38]. This inactivation phenomenon is known as the oxygen excited state and when inactivation is achieved, the onset of lipid oxidation is prevented [32]. The unsaturation of carotenoids is sensitive to oxygen, heat, light, acids, peroxides, and metals [39]. Polyunsaturated fatty acids present in vegetable oils can oxidize more easily due to the presence of their two or more double bonds, as is the case of G and P samples. However, extra virgin olive oil or sample O has only a single double bond on its structure making it less susceptible to oxidation by radicals. Faine et al., [40] reported that olive oil oxidized to a lesser degree compared to those oils that had more than two unsaturations. Figure 4 showed significant differences (*p* ≤ 0.05) and the native oils inhibited ABTS radical in a range of 92 to 97%, while the oils with lycopene inhibited it in a range of 95 to 98%. Regarding the DPPH radical, Figure 5 showed that the native oils achieved 91 to 94% of inhibition in both techniques. However, the GL, OL, and PL samples showed 2–6% of inhibition in the sonicated samples and 13–63% of inhibition in the samples subjected to agitation. These results suggested that the reduced inhibition of oils with lycopene may be due to the antioxidant molecules such as phenols, flavonoids, and carotenes present in the native oils. Olivares and other authors [41] reported some antioxidant agents could have a nucleophilic interaction with lycopene and generate prooxidation due to the possible presence of metals in the composition of native oils. Anthocyanidins are flavonoids present in grapes, these may have a prooxidant function possibly due to the conjugation between the A and B rings that influences the prooxidant action of a flavonoid initiated by Cu, while O-methylation inactivates prooxidation [42].

**Figure 6:**
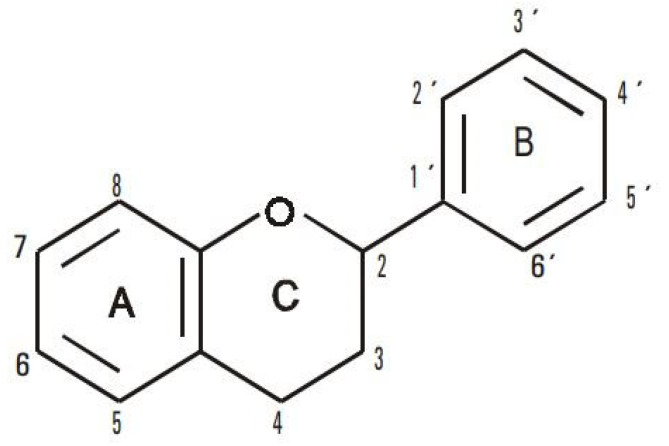
Basic structure of flavonoids showing the A and B rings, which are responsible for the prooxidant activity. Source: [42].

Figure 6 shows the conformation of the flavonoid structure. The position of the A and B rings have prooxidant activity. It is also important to mention that prooxidation can be due to the generation of reactive oxygen species (ROS) or to the neutralization of the antioxidant effects [42]. The imbalance that occurs between antioxidants (lycopene) and pro-oxidants (flavonoids from native oils) produces oxidative stress. It generates an electron blocking effect reducing the antioxidant activity of lycopene. There are two mechanisms by which antioxidant molecules such as lycopene deactivate the free radicals of flavonoids present in vegetable oils: hydrogen atom transfer and single electron transfer. Both mechanisms can occur in parallel but only one may dominate depending on the structure of the antioxidant. Lycopene can act mainly through the electron transfer mechanism because of its eleven conjugated double bonds which can interact by transferring electrons [43]. 

### 3.4. Color Determination

The color parameters L*, a*, and b* have been widely used to describe and communicate colors. L* parameter refers to Luminosity (0 = black and 100 = white); a* for red color (+ value) or green (− value). The letter b* describes yellow color (+ value) or blue (− value). According to the results shown in Table 1, the luminosity of the different native oils revealed that the O sample was lighter (L* = 41.08) than P (L* = 39.84), and the darkest sample resulted in the G treatment (39.41). In the case of the oils with lycopene, it was found that OL was the lightest (L* = 19.16), the GL sample had a luminosity of (L* = 17.05), and the PL (L* = 16.72). These samples presented greater darkening due to the red pigment from lycopene. PL sample tended to be the darkest and OL resulted in the lightest in the experiment. The values for parameter a* showed the degree of coloration of the sample of native oils, G presented a negative value (a* = −1.73) which is related to green color, while, P (a* = 0.52) and O (a* = 3.52) samples reported positive values related to red color. The samples containing lycopene tended more towards the positive side; PL with a higher value (a* = 27.10), followed by GL (a* = 27.08) and OL with lower value (a* = 26.98). Regarding values for b* parameter, the native oils resulted in values oriented to yellow color. O sample with a value about b* = 26.89. This sample resulted in the highest value. P with b* = 19.12 and G with b* = 17.36. Samples with lycopene produced b* values as follows: The highest value resulted from G sample with b* = 17.36 followed for OL (b* = 12.79) and GL (b* = 11.38). These results agree very well with those reported for Varas Condori [32] in a study to determine the antioxidant effect of lycopene extraction of tomato (*Solanum lycopersicum*) on the shelf life of flaxseed oil. CIELab color space parameters showed significant differences in flaxseed oil and flaxseed oil with lycopene being flaxseed oil with lycopene the one that showed lower brightness, higher tendency to red color, and lower tendency to yellow color. L* in flaxseed oil was 36.23 and decreased to 20.97 when lycopene was added to the oil. The value for coordinate a* was −1.40, with the addition of the lycopene to the linseed oil increased to 5.21 which is evidenced by the red color of the oil. The coordinate b* decreased from 23.43 to 4.70.

### 3.5. Microscopic Structure of Tomato Lycopene

**Figure 7:**
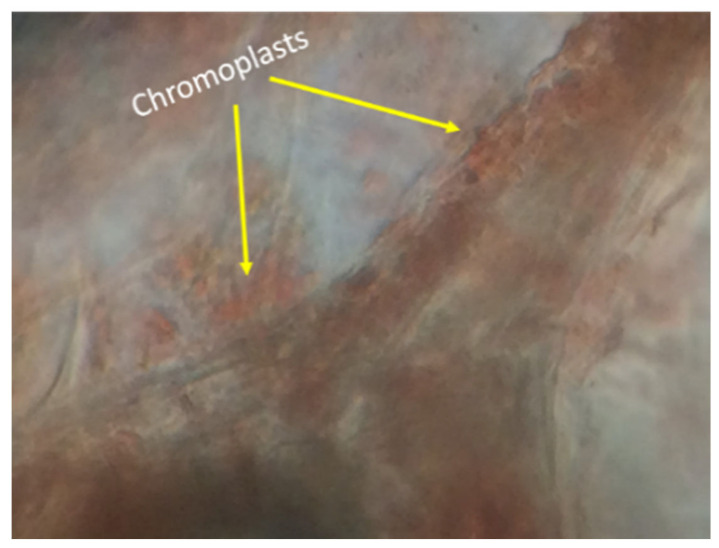
Chemical structure of tomato where chromoplasts are found.

Figure 7 shows the structure of a ripe tomato visualized with a 100× objective. The tissues showed red-colored areas belonging to chromoplasts’ presence. Chromoplasts are plastids where photosynthesis takes place [44]. These plastids are the storers of pigments such as lycopene. This carotenoid is responsible for the red coloration of tomatoes. Also, black spots were observed due to the immersion oil that was added to visualize the cells.

### 3.6. Fourier Transform Infrared Spectroscopy (FT-IR)

**Figure 8:**
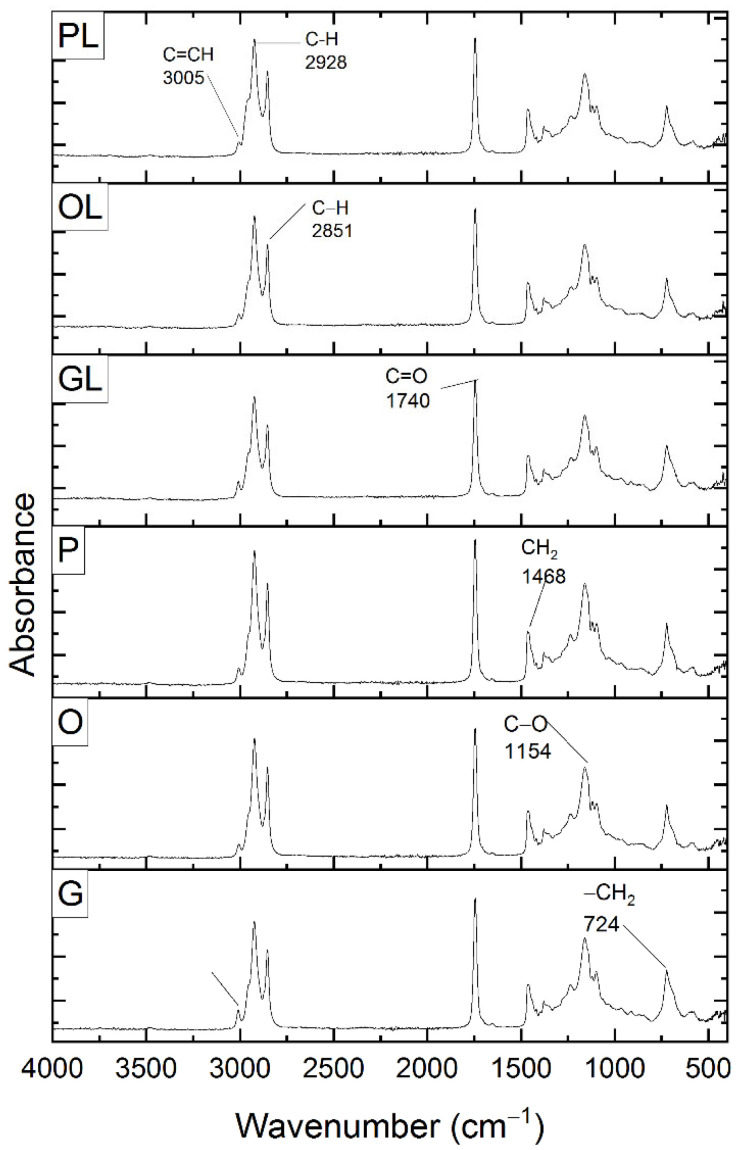
FTIR spectrum of native oils (P, O, G) and oils with lycopene (PL, OL, GL).

Figure 8 represents the studies performed on the native oils described as samples P, G, and O. It also includes the spectra of the lycopene-containing oils described in this study as PL, GL, and OL. All spectra showed the same peaks. From left to right: 3005 cm^−1^, 2928 cm^−1^, 2851 cm^−1^, 1740 cm^−1^, 1468 cm^−1^ 724 cm^−1^. Previous works [45] reported peaks in the region 3000 cm^−1^ and 2928 cm^−1^ corresponding to the stretching vibrations of C=CH. Also, the wavenumbers 2851 cm^−1^ and 1740 cm^−1^ were related to CH_2_ symmetric stretching and C=O stretching (phospholipids) respectively. The 1468 cm^−1^ peak was correlated to CH_2_ bending vibrations of lipids. From previous studies [21], lycopene was extracted from tomato waste using sunflower oil by ultrasound technique, and the C-H stretching mode corresponding to the peaks 2920 cm^−1^ and 2950 cm^−1^. These peaks were correlated with the structure of lycopene. Aghel and other authors [46] reported the vibrational wavelengths of the IR spectrum of the extracted lycopene as follows: 3100 cm^−1^ corresponding to the CH (sp2) stretching 2918.92 cm^−1^ and 2851.05 cm^−1^ corresponding to the CH (sp3) stretching. These results agree with those obtained in this research as lycopene appears in the same vibrational range. The spectra analyzed by Chemat-Djenni et al. [26] resulted from the extraction of tomato carotenoids using d-limonene from orange residues. They found stretching bands corresponding to the -C-H and = C-H functional groups at 2964.37 and 3100.08 cm^−1^. These stretches correspond to lycopene which is composed of 40 carbon atoms and 56 hydrogen atoms. Studies performed by Damayanti et al., [47] obtained FT-IR spectra from tomato lycopene samples extracted by maceration using chloroform. They observed vibrations of C-H functional groups at 2856.67 cm^−1^ and 2926.11 cm^−1^. Another study [48] reported the effect of oleic acid composition on carboxymethylcellulose-based biopolymer electrolyte. It was reported several peaks in the spectra in the region 2920 cm^−1^ and 2850 cm^−1^ assigned to symmetric and asymmetric C-H stretching of oleic acid.

### 3.7. Differential Scanning Calorimetry (DSC)

**Figure 9:**
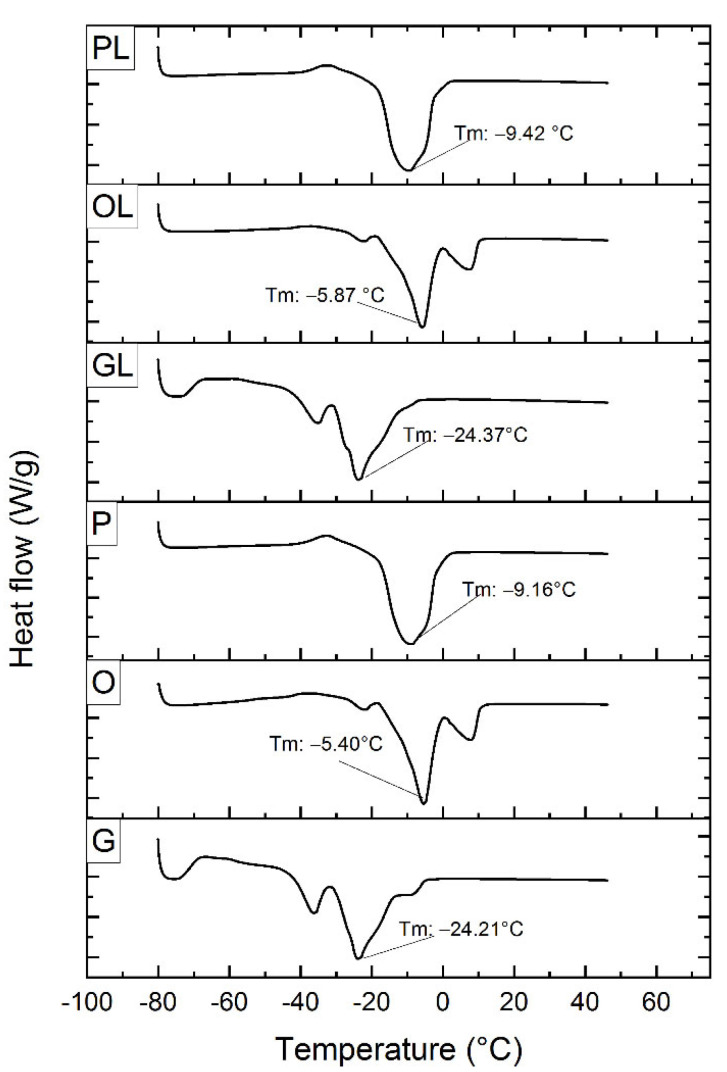
Determination of thermal properties in samples O, P, G, OL, PL, and GL, treated under ultrasonic conditions.

Figure 9 shows the differential scanning calorimetry thermograms of samples O, P, G, OL, PL, and GL subjected to sonication. In sample O, three thermal events were identified. The first region ranges from −24.49 °C to −23.69 °C, with a temperature peak at −24.48 °C. The second region showed a higher peak than the previous one with a melting point of −5.40 °C where the polyformic transformation of this oil was represented with an enthalpy of 27.82 J/g. The third region was found between 8.41 °C to 8.71 °C with a peak of 8.54 °C. Previous studies [49] reported the characterization of olive oil by differential scanning calorimetry. They found three endothermic peaks; the first region of low melting temperature ranged from −20.68 °C to −11.41 °C with a peak of −15.09 °C, and a second region starting at −11.41 °C to 3.27 °C with an endothermic peak at −3.82 °C. This point represented a polymorphic transformation of the oil. The third region of higher melting temperature started at 3.27 °C and ended at 13.19 °C with a peak at 6.23 °C. 

Regarding sample P, the thermogram was represented by two amorphous events, the highest peak presented a melting temperature of −9.16 °C and an enthalpy of 58.15 J/g. Sample G presented three events and the peak that was expressed with greater intensity presented a melting temperature of −24.21 °C with an enthalpy of 23.15 J/g. On the other hand, the samples with lycopene were expressed as follows; sample OL, showed three events; the intermediate one presented a melting temperature of −5.87 °C and an enthalpy of 30.56 J/g. Sample PL, presented two events, the most representative one with a Tm of −9.42 °C and an enthalpy of 59.80 J/g. Sample GL presented two events and the most pronounced one reported a melting temperature of −24.37 °C and an enthalpy of 33.50 J/g. It was observed that samples O, G, OL, and GL showed two peaks while P and PL showed the presence of three peaks. In previous studies in which eleven different types of oils were analyzed, it was found that the transition temperature and the shape of the Tm peaks depended on the heating rate. The most saturated triacylglycerols melted at higher temperatures than the unsaturated ones. The complexity of the thermal properties of the oils is mainly due to the triacylglycerols, so the melting temperature is not specific. The melting and crystallization of oils are important in determining their functionality when incorporated into various food products [49]. 

### 3.8. Ultraviolet-Visible Spectroscopy (UV-Vis)

**Figure 10:**
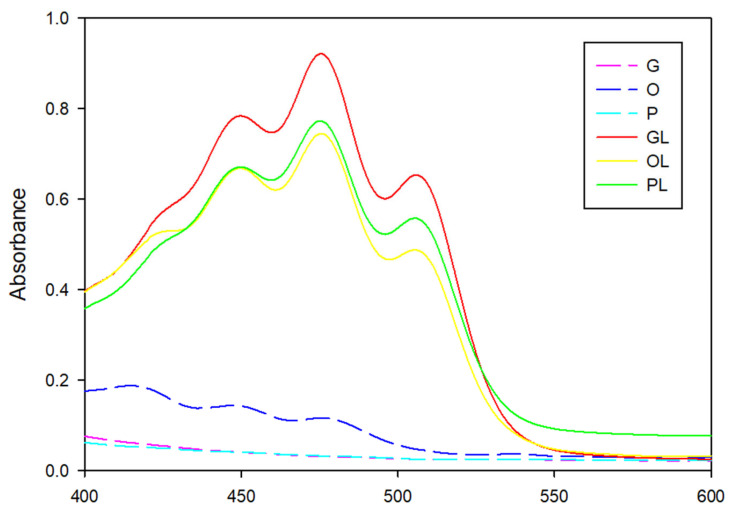
UV-Vis spectroscopy of native oils (G, O, P) and oils with lycopene (GL, OL, PL).

The spectra of native oils G and P did not show peaks in any region, while the spectra from the O sample showed slight peaks. However, samples with lycopene OL, GL, and PL showed three peaks belonging to antioxidants in the regions 448 nm, 473 nm, and 508 nm (see Figure 10). The first two peaks refer to the characteristic peaks of lycopene. The third peak belongs to other carotenoids. These results showed that lycopene was not present in native oils. Arándiga and Díaz [15] extracted lycopene with conventional solvents and upon determination by UV-Vis, they identified the presence of lycopene at 471 nm. Other authors [26] extracted lycopene with limonene from oranges. In UV-Vis absorption spectra they identified three peaks at 457 nm, 483 nm, and 516 nm. The lycopene peak was established at 483 nm. Previous works [22] performed tomato lycopene extraction with ultrasound and sunflower oil. The authors identified the peak that belongs to lycopene at 472 nm.

## 4. Conclusions

The use of high-intensity ultrasound is not a recommended technique for the extraction of lycopene due to the formation of free radicals that oxidize this carotenoid. One of the techniques recommended for lycopene extraction is the magnetic stirring technique, since this technique is less aggressive and does not form free radicals that oxidize lycopene and the compounds present in vegetable oils. Additionally, vegetable oils with high unsaturated content were also susceptible to oxidation. Peanut oil is not recommended to use as a solvent in the extraction of lycopene. However, olive oil allowed greater extraction ratios, therefore, it is highly recommended to use it as a solvent to extract this type of carotenoids. It was also concluded that the DPPH radical allowed the best inhibition of antioxidant agents due to its greater affinity with oily samples.

## Figures and Tables

**Table 1:** Color parameters L, a*, and b* in three types of native oils and oils with lycopene. Letters (**a**–**e**) represent significant differences between samples.

Sample	L	−a* +	−b* +
G	39.41 ± 0.22 **^c^**	−1.73 ± 0.04 **^a^**	17.36 ± 0.19 **^c^**
GL	17.05 ± 0.27 **^a^**	27.08 ± 0.33 **^d^**	11.38 ±0.18 **^a^**
O	41.08 ± 0.45 **^d^**	3.52 ± 1.03 **^c^**	26.89 ± 0.12 **^e^**
OL	19.16 ± 0.05 **^b^**	26.98 ± 0.01 **^d^**	12.79 ± 0.03 **^b^**
P	39.84 ± 0.20 **^c^**	0.52 ± 0.02 **^b^**	19.12 ± 0.06 **^d^**
PL	16.72 ± 0.05 **^a^**	27.10 ± 0.38 **^d^**	11.16 ± 0.03 **^a^**

## Data Availability

Data is contained within this article.
